# Socio‐economic status, executive functions, and theory of mind ability in adolescents: Relationships with language ability and cortisol

**DOI:** 10.1111/bjdp.12354

**Published:** 2020-10-14

**Authors:** Graham Pluck, Marco A. Córdova, Christine Bock, Izan Chalen, Ana F. Trueba

**Affiliations:** ^1^ Institute of Neurosciences Universidad San Francisco de Quito Ecuador; ^2^ Laboratorio de Biotecnología Vegetal Universidrad San Francisco de Quito Ecuador

**Keywords:** adolescence, cortisol, executive function, language, socio‐economic status, theory of mind

## Abstract

Socio‐economic status (SES) is linked to the development of cognitive abilities, particularly language and executive processes. It is unclear whether these represent a single or independent correlates. We studied 110 Ecuadorian youths aged 12–17 with measures of SES, language, executive function, and theory of mind (ToM), a.k.a. mentalizing. A subsample gave hair samples to estimate recent cortisol levels. Restricting analyses to reliable measures, SES was highly associated with language skill, and to a lesser extent with executive function and ToM performance. However, those latter associations were attenuated and non‐significant when language ability was controlled for statistically. Systemic cortisol levels were not associated with SES, but were significantly and negatively correlated with ToM, independent of variation in language skills. We conclude that language development underlies most of the impact of SES on executive function and ToM ability of adolescents, but that stress‐related cortisol may have an independent, direct effect on mentalizing.

**Statement of contribution**

***What is already known on this subject?***

- Language is closely linked to executive function (EF) and theory of mind (ToM) in adolescents.
- Language and EF are both closely associated with socio‐economic status (SES).
- Physiological stress, including that related to SES, is associated with language development.

***What does this study add?***

- Variability in language skill explains most of the SES‐linked variability in adolescent EF and ToM.
- Higher cortisol levels are associated with lower ToM performance in adolescents.

## Background

It is recognized that the socio‐economic situation that young people are raised in has effects on their cognitive development. With few exceptions, studies find that lower socio‐economic status (SES) is associated with lower cognitive ability (Daneri, Blair, Kuhn, & Investigators, 2019; Hackman & Farah, 2009; Pluck, Banda‐Cruz, Andrade‐Guimaraes, & Trueba, 2018). This has multiple impacts on later educational achievement and earning potential (Mood, Jonsson, & Bihagen, 2012), and health in adulthood (Cohen, Janicki‐Deverts, Chen, & Matthews, 2010).

Although SES is clearly a complex and multifactorial construct, involving, among other things, income, wealth, and power, most health‐related research only includes single measures, such as education (Braveman et al., 2005). Nevertheless, education is a strong measure of SES in general, being associated with income, occupation, and material and intellectual resources available within families (Galobardes, Shaw, Lawlor, Lynch, & Davey Smith, 2006). For adolescents, parental education level and material possessions are important indicators of SES (Elgar et al., 2016). Existing research into child and adolescent cognitive and brain development in relation to SES has tended to use either neighbourhood‐level measures (Theodoraki, McGeown, Rhodes, & MacPherson, 2020), discreet individual‐level measures, frequently parental income or education (Fatima, Sheikh, & Ardila, 2016; Lawson, Duda, Avants, Wu, & Farah, 2013; Noble et al., 2015), or composite measures including multiple factors (Corso, Cromley, Sperb, & Salles, 2016; Noble, Houston, Kan, & Sowell, 2012; Noble, Norman, & Farah, 2005). The latter approach was taken in the current research.

Cognitive studies of adopted children, who typically move from lower into higher SES situations, are informative on the causal relationship linking SES to cognitive function. A meta‐analysis has shown that although infants have relatively low IQ scores before adoption, following adoption they reach IQ levels equivalent to those of their non‐adopted peers (van Ijzendoorn, Juffer, & Poelhuis, 2005). This strongly suggests that the experience of living in a low‐SES context has a substantial negative impact on the development of cognitive functions. However, cognition is not uniformly affected. When multiple cognitive systems are examined, language ability and executive function (EF) appear to be disproportionally linked to disparity in SES in children and adolescents (Farah et al., 2006; Hackman & Farah, 2009). This is supported by structural brain imaging with adolescents showing that thickness of specifically the temporal and prefrontal cortices, thought to be key physiological substrates of language and EF, respectively, is dependent on SES (Avants et al., 2015; Lawson et al., 2013).

By EF we mean the typical functions such as response suppression, switching, and working memory: those described as ‘cool’ as opposed to ‘hot’ EF. This distinction between cool and hot EF has been proposed to summarize diversity of higher‐level functions observed in childhood and adolescence (Zelazo & Carlson, 2012). From a neuropsychological perspective, cool functions are associated with dorsal prefrontal regions, and hot functions with ventral prefrontal regions (Zelazo, Qu, & Müller, 2005). Theory of mind (ToM) ability is an example of a hot function linked to the ventral regions (Hongwanishkul, Happaney, Lee, & Zelazo, 2005; Zelazo et al., 2005). Nevertheless, it should be pointed out that, the distinction of hot and cool EF is not universally accepted. For example, Miyake et al. (2000) have proposed a tripartite division of EF, and others see EF and ToM as being closely related, yet distinct processes (Wade et al., 2018).

There is strong evidence that EF is positively associated with SES in adolescence (Fatima et al., 2016; Theodoraki et al., 2020). However, there are presently no studies reporting associations between SES and ToM ability in adolescence. One study with children aged three to four reported that task performance is positively linked to SES background (Shatz, Diesendruck, Martinez‐Beck, & Akar, 2003), but others, with children aged five to six, report no such effects (Noble et al., 2005). One reason for the dearth of reported associations and discrepant results may be that cognitive tests generally function poorly as individual difference measures (Hedge, Powell, & Sumner, 2018). This is because correlation sizes are dependent on reliability of tests used. This is a particular problem for tests of EF, as they are frequently found to have poor retest reliability with adolescent samples (Bishop, Aamodt‐Leeper, Creswell, McGurk, & Skuse, 2001; Pluck, Amraoui, & Fornell ‐Villalobos, 2019). Dang, King, and Inzlicht (2020) have argued that only cognitive tests with high reliability should be used as individual difference measures.

A further problem is whether EF and ToM should be considered as independent correlates of SES, when language ability is also highly correlated. Development of EF and ToM tend to be highly associated to the development of language skills across adolescence (Booth, Boyle, & Kelly, 2010; Valle, Massaro, Castelli, & Marchetti, 2015). In terms of directionality, a longitudinal study has reported that advanced ToM performance in early adolescence is directly linked to preschool comprehension skills, but early ToM performance is not directly linked to later comprehension skills (Ebert, 2020). This suggests that the advanced ToM ability observed in adolescence is consequent on the development of early childhood language skills. Further evidence comes from ‘natural experiments’ comparing typically developing adolescents with those who cannot develop language as efficiently due to deafness. Deaf adolescents, in addition to delayed language development (Harris & Terlektsi, 2011), typically also display worse performance on tests of non‐verbal EF (Kronenberger, Pisoni, Henning, & Colson, 2013).

How then should we interpret the oft observed co‐occurrence in youth samples of a) correlations between SES and language skills and b) between SES and EF or ToM tests? Is perhaps language development the single underlying factor? Research with kindergarten and first‐grade children has suggested that language ability mediates much of the variation in EF related to SES (Noble, McCandliss, & Farah, 2007; Noble et al., 2005), but the mediation effect may be much lower in older children (Sarsour et al., 2011), and in adolescence the association is unknown.

Based on neuroimaging evidence, it has been argued that SES‐related linguistic context may impact mainly on language development of children and adolescents, while SES‐related stress may impact mainly on EF and social‐emotional processing (Merz, Wiltshire, & Noble, 2019; Noble et al., 2012). Indeed, studies have consistently linked low SES to higher levels of general psychological stress (Matthews & Gallo, 2011), and to levels of post‐traumatic stress (Goodman, Miller, & West‐Olatunji, 2012; Pluck, Banda‐Cruz, Andrade‐Guimaraes, Ricaurte‐Diaz, & Borja‐Alvarez, 2015). The activation of the hypothalamus–pituitary–adrenal (HPA) axis that results in the secretion of hormones such as cortisol could offer a physiological mechanism through which the relationship develops. Cortisol levels can be interpreted as a proxy of psychological well‐being, even as an indicator of psychopathological conditions, (Jessop & Turner‐Cobb, 2008; Lindfors & Lundberg, 2002), an association driven primarily by the release of cortisol in response to psychological stress.

Living in low‐SES situations is associated with higher cortisol levels in children (Lupien, King, Meaney, & McEwen, 2000), and for infants and their mothers (Clearfield, Carter‐Rodriguez, Merali, & Shober, 2014). Cortisol levels are also related to cognitive functioning. One study reported that greater family instability was related to elevated cortisol patterns, which in turn was related to diminished cognitive functioning in children (Suor, Sturge‐Apple, Davies, Cicchetti, & Manning, 2015). Family instability is one of several ‘chaos’ factors that challenge child and adolescent well‐being and are particularly associated with families living in poverty (Weisner, 2010). Additionally, higher diurnal cortisol is related to a delay in language production (Saridjan et al., 2014). Suggesting a mechanism through which SES could influence language development, and perhaps other cognitive skills that may be underpinned by language, such as EF and ToM.

### Current study

As there are few studies exploring cognitive function and SES in adolescents, we attempted to address several of the aforementioned issues in a sample of people aged 12–17. We recruited a mixture of male and female participants, and from a wide range of SES backgrounds, as this avoids the attenuation of correlation strength which results from having limited variation within data sets (Howell, 1992). Recruited adolescents were assessed for both EF and ToM as well as for language development. We also ascertained the psychometric properties of the scales used and only analysed those with acceptable reliability. We then examined the correlations between SES and cognitive function, using regression‐based procedures to test the effects of covarying language ability. Finally, as an exploratory measure, and from a subsample of participants, we took samples of scalp hair and measured cortisol levels in the three centimetres closest to the scalp (which would correspond approximately to the preceding three months of growth).

Our hypotheses were:

Hypothesis 1 SES will be significantly associated with cognitive test scores, particularly language.

Hypothesis 2 Most or all of the association of SES with cognitive test scores can be explained by variation in language ability.

Hypothesis 3 SES will be associated with HPA axis activation resulting in variation in cortisol levels.

Hypothesis 4 Cortisol levels will be associated with cognitive ability.

## Method

### Design and participants

In a group of adolescents, data were collected on cognitive ability, and, as an exploratory measure, cortisol activity, to examine their linear relationships with SES as a continuous variable. In the first phase, only SES and cognitive data were collected. The second phase involved recruitment of further participants and additionally included a four‐week test–retest reliability study of the cognitive tests, as well as the collection of hair samples for cortisol assay.

We included adolescents from a wide range of backgrounds around Quito, Ecuador. Learning disabilities or sensory disability were exclusion criteria. For our main analyses on the association between cognitive ability and SES we needed at least 108 participants, based on a sample‐size estimation (*r *> .30 at α = .05 [one‐tailed] and β = .20; Cohen, 1992).

In total, 115 participants were recruited, but four were excluded for being outside of our target age range (ages 11, 11, 18, and 19), and data on SES was accidently not collected on one case. The remaining 110 participants comprised 36 females (32.72%). The reason for the predominance of males is mainly that more males volunteered. Of the final sample of 110 participants, 81 were recruited from ten different schools: five state‐run schools (*n* = 55), four private schools (*n* = 24) and one non‐governmental organization run school (*n* = 2). We ranked the neighbourhoods where each school was located based on our own knowledge: three we considered to be in socio‐economically deprived neighbourhoods (*n* = 46), two in mid‐status neighbourhoods (*n* = 9), and five in affluent neighbourhoods (*n* = 26). The remaining 29/110 participants were recruited from a Facebook appeal (*n* = 10), from a private university (*n* = 8) and from a charitable service providing residential care, education, and sports training to disadvantaged male adolescents (*n* = 11).

In terms of ethnicity, the majority 92/110 (83.64%) identified as Mestizo (mixed Indigenous American and European heritage), 13/110 (11.82%) as Afroecuadorian, 4/110 (3.64%) as Indigenous American, and 1/110 (0.91%) as Mixed‐race. The mean age was 15.0 (*SD* = 1.6, range = 12‐17). All were Spanish speakers.

As an exploratory measure, hair samples were collected from a subsample in the last phase of data collection. In this phase, 49 participants were recruited, and 32 consented to give hair samples. However, two of these were of the aforementioned excluded participants, and one hair sample could not be processed, so data were available on 29 participants: 9 females (31.03%), mean age 15.7 years (range 12–17). Twenty‐seven consecutive participants from the last phase were asked to return approximately one month later to allow estimation of retest reliability, but only 21 actually returned, mean age 16.3 (range 12–17), 8/21 female (38.10%).

### Assessments

#### Socio‐economic status

Rather than use a standardized tool from a different culture, we formed our own measure of SES, as relevant factors are quite culture specific (Braveman et al., 2005). Nevertheless, we included parental education level, and possessions (such as electronic study devices), as these are consistent indicators of adolescent SES (Elgar et al., 2016; Wardle, Robb, & Johnson, 2002). We also asked each participant about their family’s housing tenure, also a recognized marker of adolescent SES (Wardle et al., 2002), classified as owned, rented, or with extended family. Responses were used to form three dummy variables (i.e., yes or no) for each housing situation. These various items are shown in Table 1.

**Table 1:** Data collected to measure socio‐economic status

Measure	Points awarded and item	Observed frequencies/110	Included in final scale
Mother’s highest education level completed	0 = None	5 (4.55%)	Yes
	1 = Primary education	39 (35.46)	
	2 = Secondary education	37 (33.64)	
	3 = Bachelor’s degree	22 (20.00)	
	4 = Master’s degree	7 (6.36)	
	5 = Doctoral degree	0 (0.00%)	
Father’s highest education level completed	0 = None	5 (4.55%)	Yes
	1 = Primary education	29 (26.36%)	
	2 = Secondary education	34 (30.91%)	
	3 = Bachelor’s degree	26 (23.64%)	
	4 = Master’s degree	9 (8.18%)	
	5 = Doctoral degree	1 (0.91%)	
	Unknown	6 (5.46%)	
Home is owned	0 = No	37 (33.64%)	Yes
	1 = Yes	73 (66.36%)	
Home is rented	0 = Yes	26 (23.63%)	Yes
	1 = No	84 (76.36%)	
Living with extended family	0 = Yes	11 (10.00%)	No
	1 = No	99 (90.00%)	
Water supply in home	0 = No	1 (0.91%)	No
	1 = Yes	109 (99.09%)	
Electrical supply in home	0 = No	0 (0.00%)	No
	1 = Yes	110 (100.00%)	
Internet at home	0 = No	35 (31.82%)	Yes
	1 = Yes	75 (68.18%)	
Satellite TV at home	0 = No	46 (41.82%)	Yes
	1 = Yes	64 (58.18%)	
Fixed‐line phone at home	0 = No	43 (39.091%)	Yes
	1 = Yes	67 (60.91%)	
Somebody in your home has a mobile phone contract	0 = No	50 (45.46%)	Yes
	1 = Yes	60 (54.55%)	
There is a domestic maid/servant in your home	0 = No	91 (82.73%)	Yes
	1 = Yes	19 (17.28%)	
You have somewhere specific in the home for study/homework	0 = No	23 (20.91%)	No
	1 = Yes	87 (79.091%)	
You have electric devices for studying	0 = No	29 (26.36%)	Yes
	1 = Yes	81 (73.64%)	

#### Cognitive ability

##### Language

We used the Vocabulary subtest from the Wechsler Intelligence Scale for Children IV – Spanish Edition (Wechsler, 2004). This involves participants providing brief definitions of 36 words, scored from 0 to 2 points each. This samples a broad range of language skills including word recognition, semantic knowledge and oral communication skills. Performance on the WISC‐IV Vocabulary test is one of the best predictors of children’s achievements in reading, as well as written and oral expression (Wechsler, 2003).

##### Executive function

We used two tests from the Delis‐Kaplan Executive Function System (D‐KEFS; Delis, Kaplan, & Kramer, 2001). For the Design Fluency Test, in each of three trials, the participant attempts to produce as many unique four‐line designs as possible within one minute. The designs are constrained by a requirement to join dots provided on the page. The first trial has only those requirements. The second and third trials contain inhibition and switching aspects, respectively. Validity studies suggest that unique designs on trials one and two measure a similar construct – motor planning, which is separate from that of trial three – scanning (Suchy, Kraybill, & Gidley Larson, 2010). Hence, in the current research, we summed trials one and two as a measure of motor planning, and trial three was taken as a measure of visual scanning. There are two other measures of potential interest on design fluency: the number of repeated designs and the number of rule violations, both of which have been shown to be sensitive to frontal lobe dysexecutive syndrome (Cipolotti et al., 2020; Possin et al., 2012). All four measures were included as potential dependent variables, as they appear to measure different aspects of EF. The Tower Test is a problem‐solving task similar to the Towers of Hanoi and involves moving discs between three spikes. Potentially, six different performance measures can be derived (Delis et al., 2001). In a psychometric study of 264 Ecuadorian children and adolescents, which included the 110 from the current study, we showed that all but one of those six measures have poor reliability (Pluck et al., 2019). The one reliable measure is time‐per‐move ratio, consequently, that is the sole measure analysed in the current research.

##### Theory of mind

Two different measures of ToM ability were used, both available in Spanish from the test developer’s website (www.autismresearchcentre.com). The Reading the Mind in the Eyes Test (RMET; Baron‐Cohen, Wheelwright, Spong, Scahill, & Lawson, 2001), children’s version, has 28 trials in which participants are required to choose, from four possible options, the likely mental state from images of people’s eyes. Administration was as recommended by the test publisher, with no specific additional guidance on the meanings of words, however, researchers answered questions on meaning if asked by participants. The Faux Pas Test (Baron‐Cohen, O’Riordan, Jones, Stone, & Plaisted, 1999) requires participants to listen to a scenario read aloud, which is also presented to them in written form. Due to our wide age range, we extended the potential difficulty of the child version by using six child‐test scenarios plus two from the more difficult adult version. After each of the scenarios is presented, participants are required to say whether somebody said something that they should not have (i.e., a faux pas). Four of the scenarios contained a faux pas and four did not. In the four that did, the participants were asked two additional questions that probed understanding of the faux pas. There were therefore three points available in each faux pas scenario, maximum 12 points. Each of the eight scenarios also contained a control question to measure scenario comprehension. The maximum comprehension score was therefore eight points.

### Procedure

Written informed consent was taken from a parent or legal representative. Additionally, the participants themselves provided written assent, in accordance with the research ethics committee approved protocol. Data were collected in two phases. In the first, 66 participants were recruited from various schools. All were assessed in a quiet room at the school, in one‐to‐one sessions. First, the adolescents reported background demographic information, including the items on SES. Research suggests that adolescents can accurately report family SES information (Ensminger et al., 2000). Then the WISC Vocabulary, Design Fluency Test, Tower Test, RMET and finally the Faux Pas Test were administered. Each administration of the cognitive tests took around 50 min. Participants were given a gift of a coloured highlighter pen worth US$ 1.50.

In the second phase, 49 participants were recruited, to gain extra data, including on retest reliability of the cognitive assessments, and, as an exploratory measure, hair samples for cortisol assay. Data were collected in a private interview room at a university, in a school, or in the participants’ homes. After completion of the cognitive data collection, in those who assented, a hair sample was taken. This was cut as close to the scalp as possible, from the posterior vertex. For the retest element, actual mean delay from test to retest was 33.7 days (range 21–54). In this phase, participants were given a gift of a pack stationary with a value of about US$ 10. In both phases of data collection, participants were debriefed and thanked for their participation.

### Cortisol assay

Cortisol extraction was carried out using previously established protocols that included using sonication to liberate cortisol into 10 ml of methanol (Russell, Koren, Rieder, & Van Uum, 2012; Sauve, Koren, Walsh, Tokmakejian, & Van Uum, 2007). Three centimetres of each sample was taken from the base of the hair and the mass of hair used was measured. Methanol was dried by leaving the samples at fifty degrees Celsius overnight, and then, cortisol was suspended at 1 ml of PBS buffer. Cortisol measurements were carried out using a MultiscanSky at the Biotechnology Laboratory at Universidad San Francisco de Quito, an ELISA EIA kit (LDN, Nordhorn, Germany) was used. Data was transformed using a Four Parameter Logistic regression (4PL).

### Statistical analysis

The reliability of the different scales was assessed, using measures of internal consistency (e.g., Cronbach’s α), and retest reliability (Pearson correlation). Acceptable reliability for research purposes was taken as values >.60 (DeVellis, 2003). For correlational analysis, age‐adjusted cognitive test scores were produced with linear regression. Data with non‐normal distributions, based on skew and kurtosis (Kim, 2013), were transformed with the RANKIT procedure (Bishara & Hittner, 2012). Bivariate zero‐order correlations were conducted to assess the relationships of SES with cognitive test scores. Pearson statistics were used for all correlations at this stage and were one‐tailed (there is no reason to hypothesize better cognitive ability associated with SES). Strength of correlation *r* values were qualitatively interpreted as ‘small’ > .100, ‘medium’ > .200, and ‘large’ > .300 (Gignac & Szodorai, 2016). Any correlations that were significant in the zero‐order analyses were repeated as partial correlations with vocabulary covaried. As these are to confirm whether or not correlations remain (one‐directional hypotheses), analyses were one‐tailed. Finally, as we had multiple EF and ToM measures, we performed exploratory hierarchical linear regression analyses to examine whether EF scores in combination, or ToM scores, in combination, explained any additional variance in SES, beyond that explicable by age and language ability. For regression analysis, raw scores were used, but were winsorized at three standard deviations to reduce the influence of outliers. For ancillary analyses, such as scale reliability and associations with age, for regressions models, and our exploratory analyses with cortisol, the significance threshold is set at .05. However, for the main analyses on SES and cognitive function, a more stringent level of .01 is employed, in both correlations and within‐model regression effects, to control the false‐positive error rate.

## Results

### Reliability

The proportions positive for each item on the SES scale are shown in Table 1. Electrical supply to the home was reported by all participants and was thus excluded. Three items were removed (sequentially) because they had Cronbach’s item‐total correlations of less than .20: ‘living with extended family’, ‘water supply to the home’, and ‘a place to study’. The resultant version had a standardized α of .81, qualitatively a ‘very good’ internal consistency (DeVellis, 2003). The potential score range was 0–18 points with an observed range of 2–17, and a mean of 8.7 (*SD* = 3.7). Higher scores indicate higher SES. The mean score is approximately the mid‐point of the scale, no participants scored at either the minimum or maximum, and the median (8.5) and mean (8.7) are similar, suggesting that the SES scale functions well as a continuous variable in the current context.

This total SES score represented a wide range of SES backgrounds. For example, for mother’s education, 4/110 (3.64%) participants reported no formal education, in contrast, 9/110 (8.18%) reported master’s level qualifications. National estimates for Ecuador in the year 2010 were that about 2.4% of adults aged 20–24 have no education (Trading Economics, 2020, 17th August) and an estimate for master’s level education among women in 2017 was 1.2% (Index Mundi, 2020, 17th August). Maternal education level, as well as the overall measure of SES, was normally distributed. These suggest that our scale represents the overall SES range as a continuous variable.

The reliability of the cognitive measures is summarized in Table 2. The Vocabulary test had ‘very good’ internal consistency and retest reliability. For the Design Fluency Test, the motor planning trials were highly correlated and the sum score of the two trials had acceptable retest reliability. The number of repeats over the three trials also had acceptable retest reliability and was reasonably internally consistent as all three trials intercorrelated. The number of designs in the scanning trial and the number of rule violations appeared to have poor reliability and were not used in further analyses.

**Table 2:** Psychometric properties of the cognitive tests used

Scale		% Missing data	Mean (*SD*)	Internal consistency	Retest
Vocabulary	Total	0	40.5 (9.7)	.88^a^	.91
Design fluency	Motor planning	0	19.2 (16.5)	.77^b^	.76
	Scanning	0	7.0 (2.5)		.45
	Violations	0	2.8 (2.7)	.14^b^	.25
	Repeats	0	5.2 (5.0)	.41^b^	.78
Tower	Time‐per‐move ratio	13.64	3.9 (1.9)		.76^c^
Reading the mind in the eyes	Total	0	18.9 (3.6)	.61^a^	.70
Faux pas	Faux pas recognition	0.91	7.4 (3.3)	.82^a^	.79
	Control items	0.91	7.0 (1.1)	.46^a^	.22

Note

^a^ Cronbach’s alpha.

^b^ Mean intertrial correlation.

^c^ From Pluck et al., (2019). The retest statistic is the *r* value from Pearson correlation. Internal consistency is not applicable to Design Fluency: Switching and the Tower Test, as for each there is a single data point per participant.

The RMET had fairly low internal consistency and retest reliability, but still above the suggested threshold for ‘unacceptable’ (DeVellis, 2003). For the Faux Pas Test, faux pas recognition had ‘acceptable’ to ‘very good’ reliability. However, for the comprehension items, reliability scores were low. However, this is caused by a ceiling effect in the data, as the modal score was 8/8 correct. In fact, 14/21 (66.67%) of participants scored exactly the same at test and retest. Thus, the reliability estimates are unreliable. The scale is included in later analyses, mainly for use as a covariate in the analyses of faux pas recognition. Time‐per‐move‐ratio scores on the Tower Test were also used in later analyses as we have previously demonstrated their reliability (Pluck et al., 2019).

### Assessment of task performance and associations with age

Mean raw scores for all measures are shown in Table 2. As a group, the participants scored 59.56% of the maximum possible score of 68 for vocabulary. Based on Mexican normative data, for participants aged under 17 (the limit of the normative data), the mean scaled score on the Vocabulary test was 9.1 (*SD* = 3.1). Normative data are not available for the RMET. However, our observed mean score was 18.9 (*SD* = 3.6), 67.50% of the maximum possible score of 28, is comparable to a mean score on the same test of 19.6 (*SD* = 3.0) in a sample of slightly older Argentinian adolescents (Zabala, Richards, Breccia, & López, 2018). For faux pas detection, the group as a whole scored 61.67% of the maximum possible score of 12, and for comprehension items, they scored 87.50% of the maximum possible score of 8. As we adapted the Faux Pas Test, no comparison with normative data or previous studies is possible. For Design Fluency motor planning, the mean composite scaled score is 10.0 (*SD* = 2.7), and for the mean number of repeats it is 10.6 (*SD* = 2.4), based on USA normative data (Delis et al., 2001). As all of the D‐KEFS measures employed had no upper limit on scores, percentage correct is not calculable.

Correlations between the different cognitive measures (raw scores), and with age, are shown in Table 3. Notably, age was only significantly correlated with vocabulary and motor planning on the Design Fluency Test. The two different measures of ToM, RMET, and Faux Pas Test, had a large, positive and significant correlation. Similarly, the two different principle measures of EF had a large significant correlation, indicating greater motor planning associated with shorter time‐per‐move on the Tower Test. Although, motor planning, an EF, had an equivalently strong, positive correlation with RMET scores, a supposed measure of ToM. Further, vocabulary scores had large, significant correlations with all cognitive measures, bar repeats on the Design Fluency Test, indicating that better EF and ToM is generally associated with better language ability.

**Table 3:** Zero‐order correlations between the different cognitive measures (raw scores) and age

Variable	*n*	1	2	3	4	5	6	7
1. Age	110	–						
2. Vocabulary	110	.21*	–					
3. DF motor planning	110	.33***	.35***	–				
4. DF repeats	110	.04	−.03	.28**	–			
5. Towers	95	−.06	−.45***	−.34***	−.06	–		
6. RMET	109	.13	.48***	.36***	−.14	−.10	–	
7. FP detection	109	.06	.38***	.17	.01	.08	.35***	–
8. FP control	109	.16	.34***	.15	.02	−.25**	.21*	.34***

Note

DF = Design Fluency Test; FP = Faux Pas Test; RMET = Reading the Mind in the Eyes Test.

^*^ *p* < .05.

^**^ *p* < .01.

^***^ *p* < .001 (two‐tailed).

### Associations of cognitive performance with SES and cortisol

The zero‐order correlations between SES, demographic variables, and the various age‐adjusted cognitive measures are shown in Table 4. Also included in this table are correlations with cortisol scores in the subsample of 29 participants who gave hair samples. SES had no significant correlation with cortisol levels, *r* = −.12, *p* = .540. However, higher SES was associated with participants being from the ethnic majority population. This is as would be expected, as for reasons stemming from colonization in Latin America, the ethnic majority population (with European heritage) has historically had greater socio‐economic opportunity (Hall & Patrinos, 2012). Furthermore, SES was significantly and positively correlated with all but one of the cognitive measures, and with qualitatively ‘large’ effects. The exception being the number of repetitions on the Design Fluency Test. Regarding the significant correlation between SES and faux pas recognition scores, this remains significant if a partial correlation is performed with faux pas comprehension items covaried, *r* = .29, *p* = .003, suggesting that association is not caused solely by difficulty comprehending the scenarios.

**Table 4:** Zero‐order correlations of the demographic and cognitive measures (age‐adjusted scores) with SES and hair cortisol levels

Measure		SES	Cortisol
Demographics	Female^a^	.06	−.05
	Age	−.07	.05
	Minority^a^	−.24*	.24
Vocabulary	Total	.67***	−.18
Design fluency	Motor planning	.33***	−.11
	Repeats	−.06	−.02
Tower	Time‐per‐move ratio	−.36***	.01
Reading the mind in the eyes	Total	.32***	−.38*
Faux pas	Faux pas items	.38***	.10
	Control items	.38***	.03

Note

^a^ Point biserial correlation, male = 1 and female = 2, not minority = 0, minority (black, indigenous) = 1.

^*^ *p* < .05.

^**^ *p* < .01.

^***^ *p* < .001. SES with cognitive scores are one‐tailed analyses, all others are two‐tailed. Hair cortisol levels correspond to approximately the most recent three months of hair growth.

Hair‐derived cortisol levels were significantly and negatively correlated with scores on the RMET, and with a qualitatively ‘large’ effect, suggesting higher circulating cortisol in recent months was associated with worse performance. However, it should be kept in mind that this was an exploratory analysis on a small subsample of participants (*29*).

### Partial correlations controlling for vocabulary scores

The only association between cognitive function and cortisol levels in the zero‐order correlations was for scores on the RMET. This correlation, as an exploratory measure judged at the .05 threshold, remains significant, and qualitatively ‘large’ in effect, when covarying vocabulary scores, *r* = −.34, *p* = .038, suggesting that the link between test scores and cortisol is driven by ToM ability, rather than general language ability. In contrast, for the EF and ToM assessments, all correlations between task performance and SES with language controlled for are now attenuated, and non‐significant (judged at a .01 significance threshold): Design Fluency Test motor planning, *r* = .18, *p* = .030, Tower Test time‐per‐move‐ratio scores, *r* = −.10, *p* =.167, RMET, *r* = .01, *p* = .446, and for faux pas recognition scores (with faux pas comprehension scores also covaried), *r* = .12, *p* = .111.

Nevertheless, there are some residual correlations of EF and ToM with SES, even with language covaried (though not significant). Therefore, the different EF measures, and the different ToM measures, may explain additional variance in SES beyond that accounted for by language skill. This possibility was examined with two hierarchical linear regression analyses, the first focused on EF, the second on ToM. In these analyses, age was also considered as an independent variable, because raw (uncorrected for age) cognitive scores were used.

Thus, in the first hierarchical regression focused on EF, summarized in Table 5, age and vocabulary scores were entered in the first stage, producing a model that significantly predicted SES, and within which, both age and vocabulary scores were significant predictors. The addition of the two main EF scores (Design Fluency motor planning and Tower time‐per‐move ratio) did not produce a significant change in *R* ^2^. Nevertheless, within the second model, age and vocabulary scores (significance judged at *p* < .01) remained significant predictors of SES.

**Table 5:** Hierarchical linear regression to predict SES scores with EF measures

Predicting SES	Variables	Standardized β	*t*	*p*	*R* ^2^	*∆R^2^*	*F*	*p*
Model 1					.41	.41	32.00	<.001
	Age	−.23	−2.71	.008				
	Language	.67	7.99	<.001				
Model 2					.45	.04	3.00	.055
	Age	−.28	−3.27	.002				
	Language	.61	6.53	<.001				
	Tower	−.02	−0.18	.423				
	Design fluency	.21	2.33	.011				

Note

*p* Values shown for cognitive variables are one‐tailed. Significance threshold for models is .05, and for individual cognitive variables it is .01. The measure of design fluency used is valid designs on trials 1 and 2 (motor planning), design fluency repeats is not included as the two design fluency measures are partially dependent.

In the second hierarchical regression focused on ToM, summarized in Table 6, the same first step was analysed, with age and Vocabulary scores entered together, producing a significant model predicting SES, and within which, both variables were significant predictors. The addition of the RMET scores, and both measures from the Faux pas Test (faux pas detection and comprehension) significantly increased the *R* ^2^. As expected, age and vocabulary remained significant predictors of SES. However, none of the ToM measures were individually significant within the model. The increase in variance explained seemed to be driven by scores on faux pas comprehension items, however, that factor was not considered significant in our final model under our significance threshold of .01 for main hypotheses.

**Table 6:** Hierarchical linear regression to predict SES scores with ToM measures

Predicting SES	Variables within the model	Standardized β	*t*	*p*	*R* ^2^	*∆R* ^2^	*F*	*p*
Model 1					.46	.46	44.69	<.001
	Age	−.21	2.17	.004				
	Vocabulary	.69	9.40	<.001				
Model 2					.50	.04	3.02	.033
	Age	−.23	−3.23	.002				
	Vocabulary	.61	7.15	<.001				
	RMET	−.05	0.55	.291				
	Faux pas detection	.11	1.37	.086				
	Faux pas comprehension	.18	2.30	.012				

Note

RMET = Reading the Mind in the Eyes Test.

*p* Values shown for cognitive variables are one‐tailed. Significance threshold for models is .05, and for individual cognitive variables it is .01.

None of the models appeared to have problems with multicollinearity (VIF values between 1.00 and 1.51), and in all models, the residuals were normally distributed.

## Discussion

The results suggest a substantial association between cognitive ability and SES in a sample of adolescents, aged 12–17. In addition, in a subsample of 29 participants, we found a relationship between a physiological measure thought to indicate chronic levels of stress and a test of recognizing mental states from people’s eyes, thought to measure ToM. A strength of this research is that all measures used were demonstrated to have acceptable reliability in the current sample, and as such, our detected associations should also be considered reliable. This is important in view of recent warnings from psychometricians on the use of unreliable cognitive measures as individual difference scores (Dang et al., 2020; Hedge et al., 2018).

These analyses allowed us to address our four hypotheses. Firstly, we accept that SES has large correlations with measures of EF, ToM, and particularly language, in young people. Although this is not a novel observation, it is notable for being from an adolescent sample, and based on data from a non‐WEIRD country (i.e., White, Educated, Industrialized, Rich and Democratic) as described by Henrich, Heine, and Norenzayan (2010). It is also noteworthy that the strength of relation between SES and language was much higher in our study (*r* = .67) than in some previous studies such as Sarsour et al. (2011) who reported an *r* of .34. This may be due to the much wider wealth gap in low and middle‐income countries, such as Ecuador, compared to upper‐income countries.

Our second hypothesis was that variation in EF and ToM ability associated with SES may be better explained by variation in language ability. This was supported. In both partial correlation analyses, and multivariable regression analyses, when age and vocabulary scores were controlled for, test scores for neither EF nor ToM significantly varied with SES. This may, in part, be due to the close relationship between the concepts of EF and ToM (Wade et al., 2018). Indeed, we found that scores on the RMET (supposedly measuring ToM), were as closely correlated with Tower Test scores (supposedly measuring EF) as they were with our other ToM assessment, the Faux Pas Test. Similar size correlations have been previously reported for the intercorrelation of purportedly EF and ToM in adolescence (Vetter, Altgassen, Phillips, Mahy, & Kliegel, 2013).

Although several previous studies of EF and SES exist in adolescents (Fatima et al., 2016; Theodoraki et al., 2020), none have explored the role of language as a mediating factor. Our finding of no remaining relationship between SES and EF, when controlling for language skill in an adolescent sample, is consistent with a previous study in kindergarten‐age children. In that study it was reported that SES did not explain any additional variance in EF, when language skill was accounted for (Noble et al., 2005). This pattern was also shown in a group of first‐grade students (Noble et al., 2007). Thus, it has been argued that SES variation drives variation in language development, and that language development variation drives variation in EF. Nevertheless, a study with 10‐year‐old children reported no evidence that language mediated the link between SES and EF (Sarsour et al., 2011). Based on the age progressions, it could be argued that language skill only mediates the link between SES and EF in infants. However, our observation of the same effect in adolescents argues against that. Possibly the reason for the one discrepant result, reported by Sarsour et al. (2011), is that in that study the language assessment was spontaneous expression. In the current study, and the ones that did report a mediating effect (Noble et al., 2005, 2007), assessments included lexical knowledge. It may be that spontaneous expressive language measures propensity for language rather than the acquired semantic information and linguistic skill, which may link closer to domain‐general cognitive abilities, providing the link from language to EF and ToM.

We had expected that some of the relationship between cognitive ability and SES would be associated with hair‐derived cortisol levels, because, as a measure of HPA activity, cortisol levels are usually taken to indicate exposure to chronic stressful situations (Russell et al., 2012). Nevertheless, our third hypothesis was not supported: In an exploratory analysis with 29 participants, we found no significant association between SES and hair cortisol levels. A mediation of the relationship between cognitive ability and SES in our sample is therefore precluded. This finding is, in fact, consistent with one existing study which also failed to link SES variables to baseline cortisol levels, although they did report a link to cortisol reactivity (Hackman, Betancourt, Brodsky, Hurt, & Farah, 2012).

However, we did observe a relationship between chronic cortisol levels and one of our measures of ToM, the RMET, partially confirming our fourth and final hypothesis. Of the subsample analysis with 29 adolescents, those with the highest hair cortisol levels had the worst task performance. Interestingly, this was statistically independent of language skill as measured by the Vocabulary test. Taken together, our results suggest an interesting contrast: SES exerts its influence on ToM performance via language ability, while chronic physiological responses to stress, that is, cortisol release, has a direct relationship with ToM, independent of language. This is consistent with previous findings that childhood adversity reduces ToM ability (Germine, Dunn, McLaughlin, & Smoller, 2015; Nazarov et al., 2014). There is also evidence to suggest that SES can alter amygdala volumes, specifically, less parental education is associated with greater amygdala size (a known substrate on ToM; Noble et al., 2012) which could provide a mechanism to explain how both stress and SES can influence ToM.

Overall, we argue that language development is a key moderating feature of the relationship between SES and EF/ToM. Consequently, a potential intervention to improve behaviour regulation of children with low SES may be to focus on language skills, rather than EF training, which has been proposed (Neville et al., 2013). We highlight that language development may be driving the link between SES and EF, but interestingly, almost the opposite has been argued. That is, the association of reading comprehension with SES is explicable by variation in EF, and that remediation to remove SES‐based disparity within education should focus on EF training (Corso et al., 2016).

At least from our results, a case can be made for targeting language skills, with the potential to possibly improving other functions. We say this because from our data it is apparent that language moderates the relationships between EF and SES, but not vice versa.

Some limitations of the current research should be acknowledged. Our measure of SES did not include parental occupation or income, which may be important indicators. Our sample size was also fairly small, particularly for the cortisol analysis. Interpretations from that data should be made cautiously. Furthermore, our main sample was somewhat self‐selected in terms of which adolescents volunteered to participate, which also meant that approximately two‐thirds of the sample were males. Finally, various different test environments were involved. Nevertheless, we do have some strengths, particularly our use of confirmed reliable measures, and our recruitment of a sample from a wide range of SES backgrounds. Thus, we feel that we can conclude that the links between SES and EF and ToM in adolescents are substantially driven by variation in language development, which should be considered the principal cognitive skill affected by variation in SES. More tentatively, we suggest that in adolescents there may be a link between ToM or emotion recognition, with baseline cortisol levels.

## Conflicts of interest

All authors declare no conflict of interest.

## Author contributions

Graham Pluck, BSc, PhD (Conceptualization; Data curation; Formal analysis; Methodology; Project administration; Supervision; Writing – original draft) Marco A. Córdova (Conceptualization; Investigation; Methodology; Project administration; Writing – review & editing) Christine Bock (Formal analysis; Investigation; Methodology; Supervision; Writing – review & editing) Izan Chalen (Formal analysis; Investigation; Methodology; Writing – review & editing) Ana F. Trueba (Conceptualization; Formal analysis; Project administration; Resources; Writing – original draft).

## Data Availability

The data that support the findings of this study are openly available in the repository for psychological science, PsychArchives, at http://dx.doi.org/10.23668/psycharchives.2898
